# Development and validation of a nomogram for predicting the probability of new vertebral compression fractures after vertebral augmentation of osteoporotic vertebral compression fractures

**DOI:** 10.1186/s12891-021-04845-x

**Published:** 2021-11-16

**Authors:** Qiujiang Li, Xingxia Long, Yinbin Wang, Xiaomin Fang, Donggeng Guo, Jinhan Lv, Xuehua Hu, Lijun Cai

**Affiliations:** 1 Graduate School of Ningxia Medical University, Yinchuan, Ningxia China; 2 Department of Orthopedics,People’s Hospital of Ningxia Hui Autonomous Region, No. 56, Zhengyuan Street, Yinchuan, 750002 Ningxia China; 3 West China Hospital, Sichuan University, Sichuan, China

**Keywords:** Osteoporosis vertebral compression fractures, Vertebral augmentation, New vertebral compression fractures, Percutaneous vertebroplasty, Percutaneous kyphoplasty, Nomogram

## Abstract

**Introduction:**

New vertebral compression fractures (NVCFs) are adverse events after vertebral augmentation of osteoporotic vertebral compression fractures (OVCFs). Predicting the risk of vertebral compression fractures (VCFs) accurately after surgery is still a significant challenge for spinal surgeons. The aim of our study was to identify risk factors of NCVFs after vertebral augmentation of OVCFs and develop a nomogram.

**Methods:**

We retrospectively reviewed the medical records of patients with OVCFs who underwent percutaneous vertebroplasty (PVP) or percutaneous kyphoplasty (PKP). Patients were divided into the NVCFs group and control group, base on the patients with or without NVCFs within 2 years follow-up period after surgery. A training cohort of 403 patients diagnosed in our hospital from June 2014 to December 2016 was used for model development. The independent predictive factors of postoperative VCFs were determined by least absolute shrinkage and selection operator (LASSO) logistic regression, univariate analysis and multivariate logistic regression analysis. We provided a nomogram for predicting the risk of NVCFs based on independent predictive factors and used the receiver operating characteristic curve (ROC), calibration curve, and decision curve analyses (DCA) to evaluated the prognostic performance. After internal validation, the nomogram was further evaluated in a validation cohort of 159 patients included between January 2017 and June 2018.

**Results:**

Of the 403 patients in the training cohort, 49(12.16%) were NVCFs at an average of 16.7 (1 to 23) months within the 2 years follow-up period. Of the 159 patients in the validation cohort, 17(10.69%) were NVCFs at an average of 8.7 (1 to 15) months within the 2 years follow-up period. In the training cohort, the proportions of elderly patients older than 80 years were 32.65 and 13.56% in the NVCFs and control group, respectively (*p* = 0.003). The percentages of patients with previous fracture history were 26.53 and 12.71% in the NVCFs and control group, respectively (*p* = 0.010). The volume of bone cement were 4.43 ± 0.88 mL and 4.02 ± 1.13 mL in the NVCFs and Control group, respectively (*p* = 0.014). The differences have statistical significance in the bone cement leakage, bone cement dispersion, contact with endplate, anti-osteoporotic treatment, post-op Cobb angle and Cobb angle restoration characteristics between the two groups. The model was established by multivariate logistic regression analysis to obtain independent predictors. In the training and validation cohort, the AUC of the nomogram were 0.882 (95% confidence interval (CI), 0.824-0.940) and 0.869 (95% CI: 0.811-0.927), respectively. The C index of the nomogram was 0.886 in the training cohort and 0.893 in the validation cohort, demonstrating good discrimination. In the training and validation cohort, the optimal calibration curves demonstrated the coincidence between prediction and actual status, and the decision curve analysis demonstrated that the full model had the highest clinical net benefit across the entire range of threshold probabilities.

**Conclusion:**

A nomogram for predicting NVCFs after vertebral augmentation was established and validated. For patients evaluated by this model with predictive high risk of developing postoperative VCFs, postoperative management strategies such as enhance osteoporosis-related health education and management should be considered.

## Introduction

Osteoporotic vertebral compression fractures (OVCFs) mainly cause sudden severe pain, progressive kyphosis, decreased quality of life, and increased mortality [1–3]. Although patients with milder conditions may be treated with bed rest and non-steroidal anti-inflammatory drugs, many patients cannot receive non-surgical treatment due to additional health problems caused by immobilization [4]. Percutaneous vertebroplasty (PVP) and percutaneous kyphoplasty (PKP) are the most commonly used minimally invasive surgical methods to have been accepted for the treatment of OVCFs in the past decades [5, 6]. The injection of bone cement through the skin into a fractured vertebral body stabilizes the vertebral body, prevents further collapse, and achieves rapid pain relief and functional recovery [7–9].

However, with the improvement and popularization of vertebroplasty technology, there are an increasing number of related complications. Postoperative new vertebral compression fractures (NVCFs) are the most common complication, which reduce the quality of life and increase the economic burden on society [10, 11]. Preoperative and postoperative management strategies may be determined by calculating the risk of NVCFs after vertebral augmetation. Therefore, it is necessary to identify high-risk patients early and implement special preoperative and postoperative management strategies to decrease the risk of NVCFs occurring after PVP/PKP. In addition, no risk model has been built to provide a helpful individualized risk estimate for NVCFs. Hence, we developed and validated anomogram to predict the risk of postoperative vertebral compression fractures (VCFs) in our study.

### Patients and methods

#### Patients

From June 2014 to June 2018, a total of 892 patients in our institution were diagnosed with OVCFs. The inclusion criteria are as follows: 1.All patients underwent a PVP or PKP procedure. 2.The patient had obvious back pain[visual analogue scale (VAS) > 6], and limited physical activity, especially in cases of turning over or getting up. 3.The diagnosis of osteoporosis can be established if one of either A or B below is fulfilled: A. T score ≤ − 2.5 at spine/hip at Dual energy X-ray absorptiometry (DEXA); B.Sagittal L1-Hounsfield unit (HU) value≤110 on Computed tomography (CT) scan. The HU threshold with high specificity (about 90%) was used to detect osteoporosis: L1 ≤ 110HU.We measured the HU value of L2 instead of L1 when patients had moderate-to-severe vertebral fractures of L1. 4.Patients with low-energy trauma. 5.The signal change of the lumbar fracture by lumbar magnetic resonance imaging (MRI) suggesting a hyperintense T2 signal and a hypointense T1 signal, or a whole-body bone scan performed a active bone metabolism. The exclusion criteria are as follows: 1.Patients with OVCFs caused by tumor, infection, or tuberculosis. 2.Patients had coagulation dysfunction, combined systemic disease, and inability to tolerate the procedure. 3.Systemic or local infection. 4.Spinal cord compression and obvious neural symptoms such as numbness and/or muscle weakness. 5.Perform hybrid stabilizations including percutaneous posterior stabilization. 6.Unstable fracture with the involvement of the posterior elements. 7.Incomplete follow-up data. The exact indication for PVP or PKP surgery is osteoporosis vertebral compression fractures that patients with back pain were not relieved by conservative treatment, or were affected by daily life seriously. Of these 892 patients, 245 who did not meet the inclusion criteria or meet any of the exclusion criteria were excluded. During the follow-up period, 49 patients were lost to follow-up; 36 patients have died of causes unrelated to PVP/PKP. Finally, our study screened a total of 562 patients who met the criteria. From June 2014 to December 2016, 403 patients were included in the training cohort. In addition, a validation cohort of 159 patients from January 2017 to June 2018 was recruited, with the same standards as the training cohort. Patients were divided into the NVCFs group and control group, base on the patients with or without NVCFs within 2 years follow-up period after surgery. All methods are carried out in accordance with relevant guidelines and regulations. This study was approved by themedical ethics committee of the People’s Hospital of Ningxia Hui Autonomous Region (the Third Clinical College of Ningxia Medical University). All participants gave written informed consent.

#### Surgical methods

The patients received PVP/PKP in the prone position under the guidance of C-arm fluoroscopy. After local anesthesia (1% lidocaine), puncture through the pedicle, fluoroscopy lateral radiographs show that a working channel is established when the bone needle was percutaneously inserted into the posterior one-third of the fractured vertebral body, according to Jensen’s technique [12]. After the stylet was removed from the trocar, a formulated polymethylmethacrylate (PMMA) mixture was instilled, filling the fractured bone. A balloon was inflated to restore the vertebral height in the PKP before the cement injection. Aferwards, PMMA was slowly injected into the fractured vertebral body. The whole process is completed under C-arm fluoroscopy to avoid bone cement leakage. The bone cement filled the fractured vertebrae in the anterior third of the vertebral body as much as possible to form an effective mechanical column. Once bone cement leakage was detected, the bone cement injection was stopped and the puncture needle was removed.

#### Postoperative management

All patients were given oral calcium and vitamin D postoperatively. If some patients have no fever or other discomfort, they will receive intravenous infusion of Zoledronic Acid (Aclasta, 100 ml/5 mg) on the first day once a year thereafter for 3 years. Patients were reviewed on the postoperative 24 h for anteroposterior and lateral spinal radiographs and discharged 2 to 3 days after surgery. Postoperative follow-up examinations were at 1 months, 3 months, 6 months, 1 year, and 2 years in the outpatient clinic. If the patient did not return for follow-up, a telephone call was made to interview the patient. When thinking of the most pragmatic and cost-efficient method of review, anteroposterior and lateral spine X-ray were taken in an upright position from all patients. Spine MRI should be considered when patient has reappeared back pain, and a vertebral fracture is possible. MRI investigations might be more useful for making an early diagnosis of the new vertebral fracture. Participants were followed for up to 2 years with NVCFs after vertebral augmentation as the end point.

#### Assessment

Primary diagnostic criteria of NVCFs after PVP/PKP were as follows: 1.The back pain reappeared after postoperative pain relief, and limited physical activity, especially in cases of turning over or getting up. 2.The signal change of the lumbar fracture by lumbar MRI suggesting a hyperintense T2 signal and a hypointense T1 signal. Newly developed OVCFs was diagnosed as NVCFs in a different vertebral body compared to the last follow-up image on plain radiography, MRI, or bone scan. Adjacent segment fracture was defined as newly developed VCFs within 1 level above or below from the index fracture. Distant segment fracture was defined as 2 or more levels away from the index fracture.

#### Observa0074ion indicators

1. General information: gender, population, age, body mass index (BMI), and fractured vertebral body segments. (2) Surgical factors: surgical method (PVP/PKP), average bone cement dosage, bone cement leakage, bone cement dispersion, contact between bone cement and endplate, regular anti-osteoporosis treatment. (3) Comorbidities: history of diabetes, hypertension, and fractures. Bone cement has crossed the midline of the vertebral body on spine x-ray plain film is defined as good bone cement diffusion, otherwise as poor. Overcoming various obstacles in the treatment process and taking their anti-osteoporosis drugs on time according to the treatment regimen are defined as regular anti-osteoporosis treatment, otherwise as irregular. The anterior vertebral height (AVH) and Cobb angle (LKA, Cobb’s method) of the fractured vertebral body were measured before surgery and 24 h after surgery. The anterior vertebral height ratio (AVHR) was calculated as percentile of anterior vertebral height of the compressed vertebra against the mean anterior vertebral height of adjacent upper and lower vertebra. The anterior vertebral height recovery ratio (AVHRR) was defined as postoperative AVHR - preoperative AVHR. Cobb angle was defined as the angle formed by the upper and lower endplates of the fractured vertebral body. Cobb angle restoration was defined as preoperative Cobb angle - postoperative Cobb angle. A lordotic angle was assigned a positive value, and a kyphotic angle was assigned a negative value.

### Statistical analysis

Categorical variables were expressed as rates, and the chi-square test was used for comparison between groups. Continuous variables are presented as mean values ±standard deviation, and independent samples t-test or analysis of variance (ANOVA) was used for comparison between groups. The texture feature selection using least absolute shrinkage and selection operator (LASSO) regression model is used for data dimensionality reduction and feature selection. The variables with *P* < 0.05 in the univariate analysis were subjected to multivariate logistic regression analysis, and the variables with *P* < 0.05 were considered as possible predictors. Based on multivariate logistic regression analysis, a nomogram was created by R softwarea in the training cohort.

A receiver operating characteristic (ROC) curve was drawn. The area under the curve (AUC) value was calculated to evaluate the sensitivity and the specificity. Calibration plots were generated to examine the performance characteristics of the predictive nomogram. Decision curve analysis (DCA) was assessed whether the model improves forecasted net income. After that, the nomogram constructed in the training cohort was further verified in validation cohort according to the same method as above. The data were statistically analyzed using SPSS version 24(IBM Corporation, Armonk, NY, USA) and R version 3.6.1(R Foundation for Statistical Computing, Vienna, Austria). Differences were defined as statistically significant at *p* < 0.05.

## Results

### Clinical features

Of the 403 patients in the training cohort, 49(12.16%) were NVCFs at an average of 16.7 (1 to 23) months within the 2 years follow-up period (Table 1). Of these 49 patients, 18 (36.73%) were adjacent segment fractures and the other 31 (63.27%) were distant segment fractures. Of the 159 patients in the validation cohort, 17(10.69%) were NVCFs at an average of 8.7 (1 to 15) months within the 2 years follow-up period (Table 1). Of these 17 patients, 5 (29.41%) were adjacent segment fractures and the other 15 (70.59%) were distant segment fractures.

**Table 1:** Demographics and clinical characteristics of NVCFs group and Control group

	Training cohort(***n*** = 403)			Validation cohort(***n*** = 159)		
	Control group(***n*** = 354)	NVCFs group(***n*** = 49)	***P***	Control group(***n*** = 142)	NVCFs group(***n*** = 17)	***P***
**Gender,n(%)**						
Male	61 (17.23%)	10 (20.41%)	0.584	39 (27.46%)	4 (23.53%)	0.730
Female	293 (82.77%)	39 (79.59%)		103 (72.54%)	13 (76.47%)	
**Age,years,n(%)**						
< 60	22 (6.21%)	1 (2.04%)	0.003	6 (4.23%)	1 (5.88%)	0.006
60 ~ 70	140 (39.55%)	12 (24.49%)		62 (43.66%)	3 (17.65%)	
70 ~ 80	144 (40.68%)	20 (40.82%)		54 (38.03%)	5 (29.41%)	
> 80	48 (13.56%)	16 (32.65%)		20 (14.08%)	8 (47.06%)	
**Population,n(%)**						
Han	316 (89.27%)	46 (93.88%)	0.317	124 (87.32%)	13 (76.47%)	0.221
Hui	38 (10.73%)	3 (6.12%)		18 (12.68%)	4 (23.53%)	
**BMI (kg/m**^**2**^**)**	23.82 ± 3.55	23.14 ± 3.36	0.208	23.21 ± 3.86	24.13 ± 3.38	0.351
**HU values**	123.72 ± 38.44	116.12 ± 29.44	0.108	119.91 ± 36.90	111.06 ± 28.11	0.249
**History of diabetes,n(%)**						
No	320 (90.40%)	46 (93.88%)	0.429	130 (91.55%)	15 (88.24%)	0.649
Yes	34 (9.60%)	3 (6.12%)		12 (8.45%)	2 (11.76%)	
**History of hypertension,n(%)**						
No	195 (55.08%)	23 (46.94%)	0.284	81 (57.04%)	11 (64.71%)	0.545
Yes	159 (44.92%)	26 (53.06%)		61 (42.96%)	6 (35.29%)	
**History of fractures,n(%)**						
No	309 (87.29%)	36 (73.47%)	0.010	133 (93.66%)	16 (94.12%)	0.942
Yes	45 (12.71%)	13 (26.53%)		9 (6.34%)	1 (5.88%)	
**Augmentation segment,n(%)**						
T4-T9	35 (9.89%)	2 (4.08%)	0.065	17 (11.97%)	0	0.307
T10-L2	192 (54.24%)	35 (71.43%)		85 (59.86%)	11 (64.71%)	
L3-L5	127 (35.88%)	12 (24.49%)		40 (28.17%)	6 (35.29%)	
**Surgical method,n(%)**			0.396			0.325
PVP	194 (54.80%)	30 (61.22%)		74 (52.11%)	11 (64.71%)	
PKP	160 (45.20%)	19 (38.78%)		68 (47.89%)	6 (35.30%)	
**Bone cement dosage, ml**	4.02 ± 1.13	4.43 ± 0.88	0.014	3.81 ± 0.87	4.56 ± 0.77	0.001
**Bone cement leakage,n(%)**						
No	238 (67.23%)	20 (40.82%)	< 0.001	97 (68.31%)	5 (29.41%)	0.002
Yes	116 (32.77%)	29 (59.18%)		45 (31.69%)	12 (70.59%)	
**Bone cement dispersion,n(%)**						
No	102 (28.81%)	33 (67.35%)	< 0.001	38 (26.76%)	9 (52.94%)	0.025
Yes	252 (71.19%)	16 (32.65%)		104 (73.24%)	8 (47.06%)	
**Contact between bone cement and endplate,n(%)**						
No	53 (14.97%)	28 (57.14%)	< 0.001	24 (16.90%)	5 (29.41%)	0.207
Yes	301 (85.03%)	21 (42.86%)		118 (83.10%)	12 (70.59%)	
**Available anti-osteoporotic treatment,n(%)**						
No	95 (26.84%)	36 (73.47%)	< 0.001	40 (28.17%)	13 (76.47%)	< 0.001
Yes	259 (73.16%)	13 (26.53%)		102 (71.83%)	4 (23.53%)	
**Pre-op AVH, mm**	14.73 ± 3.08	14.69 ± 2.91	0.936	14.89 ± 3.26	14.74 ± 2.62	0.852
**Post-op AVH, mm**	17.66 ± 3.03	17.98 ± 2.93	0.482	17.70 ± 3.19	18.08 ± 2.17	0.630
**AVHRR(%)**	9.70 ± 3.45	10.69 ± 2.45	0.053	9.28 ± 3.03	10.72 ± 2.42	0.061
**Pre-op Cobb angle(°)**	26.97 ± 5.95	26.01 ± 4.69	0.278	28.33 ± 6.22	27.14 ± 4.25	0.444
**Post-op Cobb angle(°)**	17.32 ± 3.70	15.27 ± 4.07	< 0.001	17.64 ± 3.97	15.34 ± 5.92	0.035
**Cobb angle restoration(%)**	34.49 ± 12.33	40.55 ± 13.86	0.002	36.33 ± 14.02	43.64 ± 17.06	0.049

### Feature selection and independent risk factors of postoperative VCFs in the training cohort

**Fig. 1:**
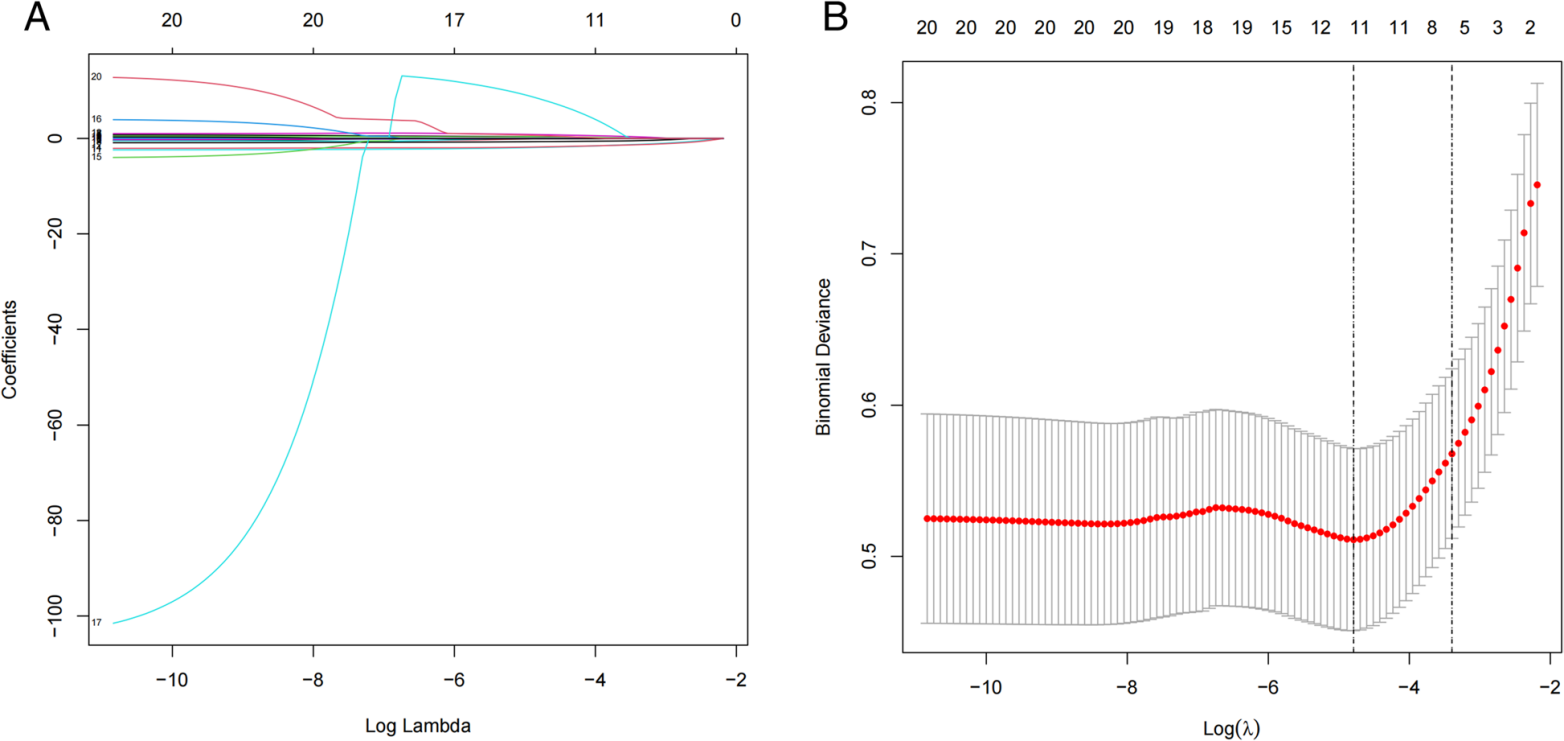
Predictor feature selection using the LASSO logistic regression model. **A** Te tuning parameter(λ) was determined in the LASSO model by using a tenfold crossvalidation and a minimum criterion. **B** The LASSO coefficient profile plot of 20 parameters was generated against the log (lambda) sequence. A vertical line was drawn at the corresponding value with Fig. A using cross-validation, and 12 nonzero parameters were selected. Notes: 1:Age, 2:Population, 3:Hypertension, 4: Fractures, 5:Bone cement dosage, 6:Anti-osteoporosis treatment, 7:Bone cement leakage, 8:Bone cement dispersion, 9:Contact between bone cement and endplate, 10:AVHRR, 11:Post-op Cobb angle, 12:Cobb angle restoration

Among the texture features (Fig. 1A, B), based on the 403 patients in the training cohort, 20 features were reduced to 12 potential predictors, which showed non-zero coefficients in the LASSO regression model. Table 1 summarizes the demographics, clinical characteristics, and imaging data of subjects in the NVCFs and control group. In the training cohort, the proportions of elderly patients older than 80 years were 32.65 and 13.56% in the NVCFs and control group, respectively (*p* = 0.003) (Table 1). The percentages of patients with previous fracture history were 26.53 and 12.71% in the NVCFs and control group, respectively (*p* = 0.010) (Table 1). The volume of bone cement were 4.43 ± 0.88 ml and 4.02 ± 1.13 ml in the NVCFs and control group, respectively (*p* = 0.014) (Table 1). The differences have statistical significance in the bone cement leakage, bone cement dispersion, contact with endplate, anti-osteoporosis treatment, post-op Cobb angle and Cobb angle restoration characteristics between the two groups (Table 1). There was no statistically significant difference between the two groups in terms of Pre-op AVH, Post-op AVH, AVHRR, Pre-op Cobb angle (Table 1). Then, multivariate logistic regression analysis demonstrated that age, bone cement dosage, bone cement leakage, bone cement dispersion, contact between bone cement and endplate, and anti-osteoporosis treatment were independent predictors of postoperative VCFs following PVP/PKP (Table 2). In addition, Table 2 shows the intercept, βcoefficient and odds ratio of the multivariate logistic regression analysis.

**Table 2:** Multivariate logistic regression analysis in training cohort

	B	SE	Wald	***P***	OR(95%CI)
**Age**			6.753	0.080	
60 ~ 70 vs < 60	1.192	1.194	0.997	0.318	3.294 (0.317 ~ 34.226)
70 ~ 80 vs < 60	1421	1.166	1.486	0.223	4.143 (0.421 ~ 40.743)
> 80 vs < 60	2.339	1.199	3.805	0.051	10.374 (0.989 ~ 108.842)
**Fractures (yes)**	0.812	0.545	2.218	0.136	2.253 (0.774 ~ 6.582)
**Bone cement dosage**			9.377	0.025	
3 ~ 4 ml vs < 3 ml	0.023	0.553	0.002	0.967	1.023 (0.346 ~ 3.028)
4 ~ 4.5 ml vs < 3 ml	−0.765	0.902	0.719	0.397	0.465 (0.079 ~ 2.727)
> 4.5 ml vs < 3 ml	1.185	0.525	5.097	0.024	3.270 (1.169 ~ 9.148)
**Anti-osteoporosis treatment (no)**	2.209	0.452	23.868	< 0.001	9.105 (3.753 ~ 22.086)
**Bone cement leakage (yes)**	1.140	0.424	7.227	0.007	3.128 (1.362 ~ 7.183)
**Bone cement dispersion (no)**	0.664	0.432	2.362	0.024	1.942 (0.833 ~ 4.528)
**Contact with endplate (no)**	2.168	0.454	22.845	< 0.001	8.743 (3.594 ~ 21.272)
**Post-Cobb**	−0.065	0.063	1.069	0.301	0.937 (0.828 ~ 1.060)
**Restoration-Cobb**	1.351	1.870	0.521	0.470	3.860 (0.099 ~ 150.831)
**Constants**	−6.013	2.039	8.695	0.003	

### Draw a nomogram through the training cohort

**Fig. 2:**
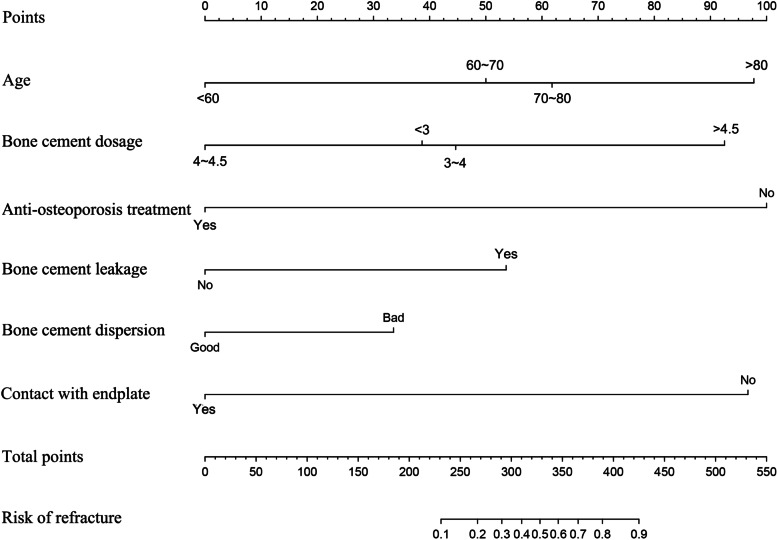
The developed nomogram of new vertebral compression fractures after vertebral augmentation. Mark the values at each factor axis, acquire the corresponding points at the points axis, and sum up the points of all factors. Mark the total points on the total point axis and draw a perpendicular line towards the risk of leakage axis. The value on the bottom line gives the probability of the cement leakage. Note: The nomogram of postoperative VCFs was developed in training cohort, including age, bone cement dosage, bone cement leakage, bone cement dispersion, contact between bone cement and endplate, and available anti-osteoporosis treatment

The model was established by multivariate logistic regression analysis to obtain independent predictors (Fig. 2). Among them, six predictors were used: age, bone cement dosage, bone cement leakage, bone cement dispersion, contact between bone cement and endplate, and anti-osteoporosis treatment. Each predictor is located on the relevant axis, and a straight line is drawn to the vertex axis to obtain a point based on the predictor. The total score is calculated by adding all the scores obtained from each predictor. The final sum is placed on the total points axis, and a straight line is drawn from there to get the non-fixed probability.

### Verification of nomogram

**Fig. 3:**
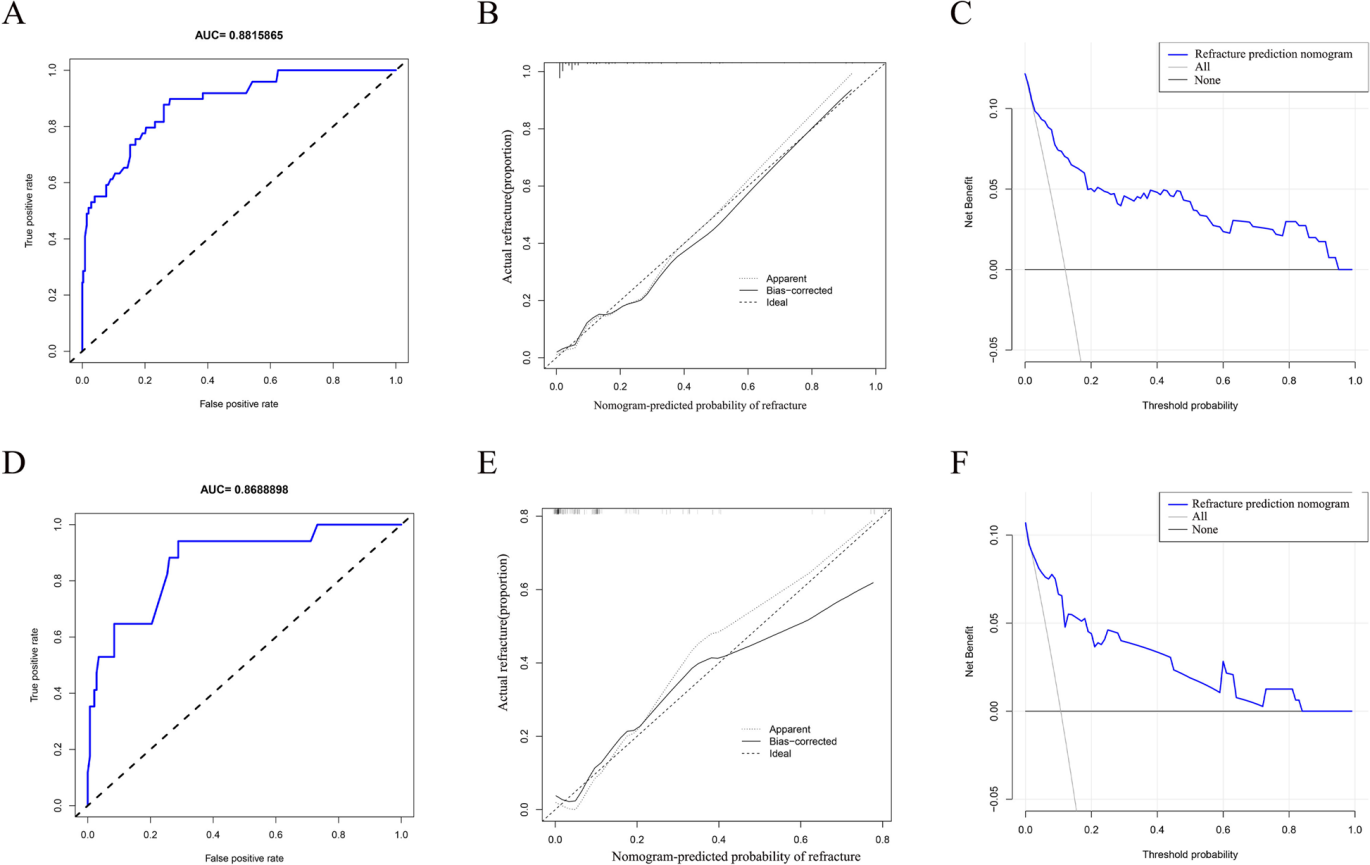
The receiver operating characteristic curves with corresponding area under the curves of nomogram and independent predictors in training cohort (**A**) and validation cohort (**D**). Calibration of the nomogram in training cohort (**B**) and validation cohort (**E**). The lines in the figure represent the apparent value, the bias corrected value, and ideal value. The apparent and the bias corrected values are close to each other, which means the nomogram has a good predictive performance. Decision curve analysis for nomogram prediction of risk of postoperative VCFs in training cohort (**C**) and validation cohort (**F**). The y-axis shows the net benefit: x-axis shows the threshold probability. The blue line represents the net benefit of our nomogram. The oblique gray line indicates the hypothesis that all patients had positive VCFs. The black horizontal line represents the hypothesis that no patients had positive VCFs

Based on the results of multivariate logistic regression analysis, we construct the nomogram with age, bone cement dosage, bone cement leakage, bone cement dispersion, contact between bone cement and endplate, and anti-osteoporosis treatment (Fig. 2). In order to verify the accuracy of the nomogram, we performed internal and external validation through concordance indices (C index) and calibration curve. The C index of the nomogram was 0.886 in the training cohort and 0.893 in the validation cohort, demonstrating good discrimination. In the training cohort (Fig. 3A), ROC showed that the obtained model has a fairly good discrimination ability, with an AUC of 0.882 (95% confidence interval (CI), 0.824–0.940), indicating that it is accurate to predict the risk of NVCFs after vertebral augmentation. The calibration curve shows that the prediction of the nomogram is highly consistent with the actual observation (Fig. 3B). In the validation cohort, the AUC of the model was 0.869 (95% CI: 0.811–0.927) (Fig. 3D). The same calibration curve shows the prediction and observation of the probability of NVCFs after vertebral augmentation (Fig. 3E).

### DCA curve analysis

In the training cohort, DCA showed that if the threshold probability of patients and doctors is greater than 2% and less than 96%, using the nomogram to predict the risk of NVCFs is more beneficial than the program (Fig. 3C). Similarly, in the validation cohort, DCA showed that if the threshold probability of patients and doctors is greater than 2% and less than 84%, using nomogram to predict the risk of NVCFs has a net benefit (Fig. 3F). In general, the nomogram is feasible and can be used to make reasonable predictions.

## Discussion

Vertebral augmentation, as a minimally invasive technique, is considered a treatment option for OVCFs. NVCFs is the most common complication reported in patients with OVCFs and remains a vexing problem for both clinicians and patients.

However, there are many factors may influence NVCFs development, including sex, age, BMI, BMD, diffusion of bone cement, and volume of cement factors. Through multivariate logistic regression analysis, we found that advanced age, excessive(> 4.5)/insufficient(< 4 ml) bone cement volume, bone cement leakage, poor bone cement dispersion, bone cement not contacting the endplate, and without available anti-osteoporotic treatment are the independent risk factors of NVCFs after vertebral augmentation for OVCFs. Then, we develop a nomogram based on six independent predictors. Nomograms are visual statistical models that can better improve clinical decision-making [13–15]. Our study integrates the predictors of demographics, surgery, and treatment characteristics into an easy-to-use nomogram, using only six easily available variables to predict the risk of NVCFs after vertebral augmentation and provides a relatively accurate prediction tool for the risk of postoperative VCFs. Meanwhile, the results of external verification also show positive discriminative capacity and calibration ability, and the high AUC values indicate that the nomogram may be widely and accurately applied.

In line with previous studies [16], six independent variables were identified as predictors of postoperative VCFs. The risk factors of NVCFs after vertebral augmentation have been revealed in numerous retrospective studies with inconsistent results [17, 18]. The reasons for the significant difference in the results may be related to the surgical skills, postoperative anti-osteoporosis treatment, distinctions in medical levels among different countries, and incidence of osteoporosis [19, 20]. There is no higher-quality research because that it is difficult to achieve homogeneity in the research. Consistent with the results reported by Zhang [16], of the 403 patients in the training cohort, 49(12.16%) had NVCFs.

Many scholars agree that the surgical factors and progression of osteoporosis are correlated with postoperative VCFs [17]. Regarding age, our study demonstrated a higher proportion of elderly patients older than 80 years in the NVCFs group (32.65%) than in the control group (13.56%). In addition, multivariate analysis revealed that age was an independent risk factor for NVCFs after vertebral augmentation. With an increase in age, the risk of NVCFs also significantly increased [21–23]. Furthermore, more than 20% of patients with osteoporosis have an increased risk of sustaining a second fracture after the first fracture without available anti-osteoporotic treatment. Available anti-osteoporotic treatment has the potential to reduce the progression of osteoporosis and prevent the occurrence of NVCFs [24, 25]. A 3-year follow-up study reported conducted Bawa et al .[26] showed that available anti-osteoporosis treatment may significantly reduce the incidence of postoperative VCFs. Multivariate analysis revealed that available anti-osteoporotic treatment is an important risk factor for NVCFs after vertebral augmentation. Therefore, anti-osteoporotic treatment should be a routine treatment in patients with OVCFs who undergo vertebral augmentation, with the aim of decreasing the occurrence of NVCFs.

Kwon et al .[27] reported that surgeons should inject as much bone cement as possible during surgery and that efficacy is optimal when the injected bone cement volume achieves 27.8% of the volume of the fractured vertebra. In addition, other studies have also recommended the use of a greater volume of cement during the surgery based on the observation that the degree of pain relief is positively related to the volume of bone cement [28, 29]. However, these studies did not consider the long-term results or NVCFs during follow-up. Excessive bone cement volume may increase the strength of the vertebral body and increase the risk of NVCFs and bone cement leakage. Zhu et al .[30] found that the volume of bone cement in the thoracic vertebrae should be less than 3.5 mL, and in the lumbar vertebrae, this should not exceed 4 mL, which may effectively prevent bone cement leakage to a certain extent. The results of our study showed that excessive(> 4.5) and insufficient(< 4 ml) bone cement volume increase the occurrence of postoperative VCFs. When the volume of bone cement is controlled at 4-4.5 mL, the risk of postoperative VCFs is the lowest. Although the results of our study confirm that an appropriate volume of bone cement effectively reduces the risk of postoperative VCFs, individualized treatments should be implemented for patients with fractures of varying severities.

The differences between the NVCFs and control group in the postoperative Cobb angle and restoration of the Cobb angle were statistically significant. The postoperative Cobb angle in the NVCFs group (15.27 ± 4.07 °) was lower than that in the control group (17.32 ± 3.70 °). Kang et al .[31] found that excessive Cobb angle restoration was a risk factor for NVCFs after PVP. One probable reason is that a larger Cobb angle would result in numbness in the vertebral internal structure and imbalanced stress, which would have led to imbalance stress of the sagittal spine, increasing the risk of refracture. However, multivariate analysis revealed that the postoperative Cobb angle and restoration of Cobb angle were not risk factors because they were closely associated with the surgical operation and bone cement dosage. This is in line with a previous study reported by Li et al .[32].

The present study found that poor bone cement dispersion in the vertebral body and failure to contact the upper and lower endplates are risk factors for postoperative VCFs. A biomechanical study reported by Chevalier et al .[33] showed that the balloon expansion effect of vertebral augmentation squeezes the loose cancellous bone to the surroundings to form a cavity, causing the bone cement to be blocked by the surrounding dense trabecular bone, reducing the dispersion of bone cement. Some researchers have reported that the bone cement diffusion may be evaluated by based on whether the bone cement could cross the midline of the vertebral body on spine x-ray plain film [34]. It was found that the clinical effect of bone cement crossing the midline of the vertebral body was better than that of bone cement on one side. When the bone cement is sufficiently diffused to contact the upper and lower endplates, the load on the vertebral body can be transferred, which greatly enhances the strength and stiffness of the vertebral bodies. On the contrary, the strength and stiffness of the vertebral bodies are significantly reduced when the bone cement only contacts one endplate. Therefore, effective bone cement dispersion and cement contact with both the upper and lower endplates decreases the occurrence of NVCFs after vertebral augmentation.

Our study established a nomogram model based on a large cohort, and successfully verified it in a validation cohort. All variables included in the nomogram are easy to determine. By calculating the points for each of the six variables, a spine surgeon can easily estimate the risk of NVCFs after a surgery. Based on the evaluation results, preoperative and postoperative management strategies can be implemented to decrease the risk of NVCFs. Similarly, for low-risk patients, some preventive measures may be reduced to reduce the economic burden.

Our research also has some limitations. First, this was a retrospective study; therefore; there may be an inherent selection bias. However, we included as many preoperative and surgical factors as possible based on a large sample to minimize deviation. Second, although this nomogram has been verified in the validation cohort, we must realize that the incidence of postoperative VCFs reported in different hospitals, regions, and countries differs, which may limit this nomogram to a small number of hospital applications. Third, because some data were lost in the retrospective study, bone mineral density was not considered in the multivariate regression analysis. Fourth, 4 of the 6 identified parameters were determined intraoperatively, although many pre- and intraoperative parameters were incorporated in the current study. It is inappropriate to assess the risk of postoperative VCFs before surgery, and the eventuality of augmenting adjacent segments must be made intraoperatively. The sensitivity and specificity of the nomogram may be further improved through multi-center retrospective validation studies or prospective randomized clinical trials, which will provide high-level evidence for future clinical applications.

## Conclusion

Our study found that advanced age, the volume of bone cement (< 4 or > 4.5 ml), bone cement leakage, poor bone cement dispersion, non-contact betwen bone cement and endplate, and irregular anti-osteoporotic treatment are the independent risk factors of NVCFs after vertebral augmentation of OVCFs. The nomogram containing the above six predictors can accurately predict the risk of VCFs after surgery.

## Data Availability

Please contact the corresponding author for data requests.

## Abbreviations

OVCFs: Osteoporotic vertebral compression fractures
NVCFs: New vertebral compression fractures
VCFs: Vertebral compression fractures
PVP: Percutaneous vertebroplasty
PKP: Percutaneous kyphoplasty
PMMA: Polymethylmethacrylate;
MRI: Magnetic resonance imaging
BMI: Body mass index
AVH: Anterior vertebral height
AVHR: Anterior vertebral height ratio
AVHRR: Anterior vertebral height recovery ratio
ANOVA: Analysis of variance
CI: Confidence interval
LASSO: Least absolute shrinkage and selection operator
AUC: Area under the curve
DCA: Decision curve analysis
ROC: Receiver operating characteristic curve
NSAID: Non-ateroidal anti-inflammatory drug
VAS: Visual Analogue Scale
DEXA: Dual energy X-ray absorptiometry
HU: Hounsfield unit
CT: Computed tomography
